# Foreign Summary

**Published:** 1838-01-17

**Authors:** 


### FOREIGN SUMMARY.

*Curious case of Simultaneous Dislocation of both Thighs.*—A sailor was sitting astride a plank, when a wave suddenly forced him up against a cross beam, which struck his back violently, while the plank was still between his legs. The poor fellow was lying on his back, when Dr. Sinogowitz was summoned to his assistance. Both limbs were quite motionless, and evidently much deformed from their natural figure. The thighs were separated the one from the other, and could not be approximated; the trochanters were much lower and less prominent than usual, and the muscles of the hips over them were in a state of extreme tension. The body was bent immovably forwards and downwards upon the thighs; the knees were moderately flexed, and the toes were not turned either inwards or outwards. The diagnosis, therefore, was that the heads of both of the thigh bones were dislocated downwards and inwards. The reduction was effected in the following manner. The pelvis being secured by two assistants, the surgeon took his place between the limbs of the patient, and having put a towel round the right thigh above the knee, he passed the noose of it over his own neck. Extension was then made by means of a towel made fast above the ancle, and inclined a little to the left side, and while this was steadily continued, Dr. S. lifted the head of the bone, and directed it upwards and somewhat outwards, by raising and stretching out his head with all his power. It slipped into the socket without any noise. The left limb was then reduced in nearly a similar manner. The mobility of the limbs was almost immediately restored, at least in the horizontal position; but several months elapsed before the patient could walk with any degree of ease. The tediousness of the recovery was owing in a very great measure, to the severe injury of the lumber vertebræ, which he sustained at the time of the accident. For three weeks, the sphincters of the bladder and rectum were quite paralyzed.—*Preussische Medicin. Zeitung.*

*Successful case of Amputation at the hip joint.*—A country woman, 25 years of age, was admitted in August, 1832, into the surgical hospital at Erlangen. From the age of 12 to 15 years she had had a fistulous abscess, situated immediately above the internal condyle of the left thigh. When this abscess healed, her health was remarkably good, and continued so until about a twelvemonth previous to her admission into the hospital. At that time the whole of the left lower extremity became swollen and painful. Two abscesses formed above the condyles, and, when they broke, discharged a large quantity of pus. The patient became hectic, amputation was proposed, and she at length submitted to it. The knee was permanently bent; it was much enlarged, and extremely tender on pressure. On introducing a probe along the fistulæ, the bone was found to be carious and necrosed. The limb was amputated in the upper third of its extent. On sawing through the bone, it was found that it was unsound. On detaching the muscles from it for 2 or 3 inches, M. Jaeger discovered that the trochanters were affected with caries. He now determined to disarticulate the head of the femur. This was done without much difficulty.—The acetabulum fortunately was quite healthy. Very little blood was lost in this second operation. The quantity of soft parts was much greater than was necessary to form a good stump; but M. Jaeger was unwilling to subject his patient to more suffering, as already she was in a weak and fainting state. He therefore united the wound with a few stitches, leaving its lower angle free, to permit the issue of any discharges. The stump, enveloped in linen wet with cold lotion, was laid upon a cushion of bran, and no bandages were applied. On the third day after the operation, three of the stitches, and also the strips of plaster were removed, in consequence of the lips of the wound having become somewhat swollen and painful, and dressings of simple cerate were applied, and retained in their place by a few turns of a roller. Light cordials and nourishing broth were allowed, in small quantities at a time, as the vital powers were rather low and apt to flag. The upper angle of the wound having assumed somewhat of a livid hue, it was frequently bathed with aromatic fomentations, sharpened a little with pyroligneous acid. Under the application of these, continued for ten or twelve days, this disagreeable appearance gradually subsided, and at the same time the character of the purulent discharge was much improved. By the end of the fourth week the wound was nearly cicatrised; the general health of the patient improved rapidly; and menstruation, which had ceased for upwards of a twelvemonth, returned on the seventh week after the operation.

The state of the amputated limb was as follows, The subcutaneous cellular tissue around the knee was thickened to a very considerable extent. All the parts of the joint were carious; the femur was thickened and uneven; and on its posterior surface were three openings, through which might be felt the sequestrous bone. On examining the bone, where it was sawn through at first, it was found to be internally carious; and this unhealthy state might be traced upwards as high as the two trochanters. The head and neck of the femur were internally soft, and very vascular.

Professor Jaeger, in discoursing on the preceding case, attributed its successful termination in part to the simple manner in which the bone was disarticulated. He is inclined to recommend surgeons to consider more attentively than they have done hitherto the plan which he followed—viz. to perform the circular operation first, and then to extirpate the head of the bone from its socket. The state of the patient's health too was favorable to the cure. Naturally of a sound constitution, there was precisely that degree of feebleness, which keeps down inflammatory reaction, and which thus is decidedly favorable to recovery after great surgical operations. The simple mode of dressing the wound at first with cold fomentations, and the abstaining from all compression with bandages, and so forth, is strongly recommended.—*Schmidt's Iahzbucher.*

---

*Death of M. Alibert.*—Died at Paris, early in November last, Baron Alibert, Professor of Materia Medica in the Faculty of Medicine of Paris, and Member of the Royal Academy of Paris. His principal work is: A complete treatise on diseases of the skin, being a *clinique* of the Hospital Saint Louis.

---

*Notice concerning the poisoning of seven horses by the arseniate of Potash, by M. Bouley the younger.*—By an unfortunate accident these horses took with their corn some arseniate of potash. Four of them died in from thirteen to seventeen hours, the remaining three, to which was given *the precipitate*, which was formed by volatile alkali, from a solution of sulphate of iron, long previously exposed to the air, lived respectively thirty two hours, fifty four hours, and nine days. The symptoms were violent colic and diarrhoea; and appearances of inflammation were discovered after death, in the stomach, intestines, and bladder. Numerous ecchymoses were seen in the basis of the left ventricle of the heart, but no traces of poisoning were discoverable in the contents of the stomach or bowels.

As the antidote employed seemed to have some effect in prolonging life, the author offers, in a subsequent paper, some observations on the efficacy of the *hydrate of the per oxide of iron*, as an antidote to arsenic.

The experiments were seventeen in number, and the following is the result. He found both the hydrate of the per oxide of iron, and the sulphate of iron, in no ways serviceable against the arseniate of potash. A horse requires not less than two ounces of the white oxide of arsenic to destroy him, and death happened in the three instances mentioned, in 45, 48, and 52 hours after its administration.

Hydrate of per oxide of iron acted as a complete antidote to white oxide of arsenic, in four cases, when given in doses, of 32 times the quantity exhibited.

The same effect happened, when the antidote was administered two and four hours after the poison; but when it was delayed to twenty four hours, and some of the first appearances of poisonous operation began to exhibit themselves, the prophylactic agency was entirely lost and death speedily took place. In one of these cases in which the horse was destroyed 62 hours after the poison, and its antidote was given, no trace of arsenic, or its neutralized combination, was to be discovered by the most careful analysis.

*Acetate of lead in Spasmodic Cholera.*—In a letter to the editor of the London Medical Gazette, Dr. Graves, of Dublin, strongly recommends the free employment of acetete of lead in Asiatic Cholera, from an extensive and successful trial of this medicine. With all the received methods of treatment in this disease Dr. G. had failed to produce any beneficial effects; but from "the frequent opportunities he had of admiring the efficacy of this salt in checking profuse alvine discharges," he determined to give it a trial in this more formidable affection. The result was that "every hour gave additional proofs of the efficacy of the remedy." Dr. Graves' formula is as follows:

℞. Acetat. plumb. ℈j; opii, gr. j. M. Ft. secund. art. mas. in pil. xij. div.

"The premonitory diarrhœa has almost invariably stopped by taking one of these pills every hour, and as the stools become less frequent, every third or sixth hour, according to circumstances. When the vomiting, spasms, state of collapse had begun, it was necessary to give a pill every quarter of an hour; after a couple of hours the effect of the pills became perceptible, in a diminution of the serous evacuations upwards and downwards—then the pills were given only every hour; and as the symptoms yielded they were given less and less frequently, and could, in general, be laid aside altogether before twenty-four hours. In some it was found necessary to give the acetate of lead in solution, combined with a little vinegar, and minute doses of the acetate of morphia. . . . . . . . . . . . . I cannot conclude without imploring the profession, in every part of the world where cholera prevails, to give my plan of treatment a fair trial, for I feel confident of its efficacy."—*London Med. Gaz. Oct.* 1837.

---

*Case of severe laxation of the knee, with reflections on the nature of this accident, and on the mechanism of the cause which produced it; by Baron Larrey.*—In all cases of this kind the author is of opinion, that an anchylosis of the joint is the only favourable issue; and that where there is extensive laceration of the ligaments, complicated with an external wound, and protrusion of the head of a bone, as in the present instance, immediate amputation should be had recourse to. The patient was in this case averse to the removal of the limb, at the time that it was first thought desirable; and though he at length submitted to it, it was not till the 21st day after the accident. For some days he went on well; but symptoms of pneumonia, with which he had been previously affected, came on about 14 days after the operation, which being combined with cerebral inflammation, soon carried him off.

